# Fabricating Water Dispersible Superparamagnetic Iron Oxide Nanoparticles for Biomedical Applications through Ligand Exchange and Direct Conjugation

**DOI:** 10.3390/nano6060100

**Published:** 2016-05-26

**Authors:** Tina Lam, Pramod K. Avti, Philippe Pouliot, Foued Maafi, Jean-Claude Tardif, Éric Rhéaume, Frédéric Lesage, Ashok Kakkar

**Affiliations:** 1 Department of Chemistry, McGill University, 801 Sherbrooke Street West, Montreal, QC H3A 0B8, Canada; tina.lam@mail.mcgill.ca (T.L.); pramod.avti@gmail.com (P.K.A.); 2 Department of Electrical Engineering, École Polytechnique de Montréal, C.P. 6079 succ. Centre-ville, Montreal, QC H3C 3A7, Canada; ph.pouliot@gmail.com; 3 Research Center, Montreal Heart Institute, 5000 Bélanger Street, Montreal, QC H1T 1C8, Canada; maafi.foued@gmail.com (F.M.); jean-claude.tardif@icm-mhi.org (J.-C.T.); 4 Department of Medicine, Université de Montréal, Montreal, QC H3T 1J4, Canada

**Keywords:** superparamagnetic iron oxide nanoparticles, nanoparticle ligand functionalization, contrast agents, magnetic resonance imaging, cell internalization

## Abstract

Stable superparamagnetic iron oxide nanoparticles (SPIONs), which can be easily dispersed in an aqueous medium and exhibit high magnetic relaxivities, are ideal candidates for biomedical applications including contrast agents for magnetic resonance imaging. We describe a versatile methodology to render water dispersibility to SPIONs using tetraethylene glycol (TEG)-based phosphonate ligands, which are easily introduced onto SPIONs by either a ligand exchange process of surface-anchored oleic-acid (OA) molecules or via direct conjugation. Both protocols confer good colloidal stability to SPIONs at different NaCl concentrations. A detailed characterization of functionalized SPIONs suggests that the ligand exchange method leads to nanoparticles with better magnetic properties but higher toxicity and cell death, than the direct conjugation methodology.

## 1. Introduction

Magnetic resonance imaging (MRI) is a sensitive biomedical tool that has been used extensively to detect various pathologies ranging from cardiac complications [1,2,3], stem cell tracking [4,5,6], to cancer treatment and theranostics [7,8,9]. Designing superparamagnetic iron oxide nanoparticles (SPIONs) as contrast agents for MRI, constitutes a topical area of research, as they possess good negative contrast capability and low cytotoxicity. Bare SPIONs oxidize and aggregate readily, leading to poor magnetic properties and undesired agglomeration during perfusion [10,11,12]. In order to impart high magnetic saturation and decreased agglomeration in solution, capping agents based on carboxylic acid end groups, such as oleic acid (OA), sodium oleate, and citric acid (CA), have been employed [13,14]. It is, however, not possible to use OA coated nanoparticles as MRI probes, because of their hydrophobic nature [15]. Thus, it is necessary to impart water-solubilizing properties to SPIONs in order to improve circulation time. Many studies have explored the physico-chemical properties and biological fate of SPIONs, which are rendered water-soluble after a ligand exchange protocol. It involves stabilization of SPIONs with a long chain hydrophobic molecule first [14], followed by the introduction of a hydrophilic ligand [16]. Such a capping process is dynamic, and in general, stronger chelators such as phosphonate and catechol-based ligands have higher affinity towards SPIONs surface [16,17,18,19,20,21].

Ligand exchange with polyethylene glycol (PEG) [22,23,24] or smaller oligomers, such as tetraethylene glycol (TEG) and triethylene-glycol (TREG), is widely used to confer aqueous solubility to OA coated SPIONs. In a study by Davis *et al.* [25], ^14^C radio-labelled oleic acid was used to cap bare colloidal SPIONs synthesized via thermal decomposition. Exchange reactions were then carried out using ligands terminated with amines, carboxylic acids, and phosphonates. Radioanalytical determination and quantification of ^14^C were used to determine, if all the radio-labelled OA had detached from the surface of SPIONs. Although significant loss of OA was observed, residual trace amounts were still present after ligand-exchange. Recently, OA and sodium oleate capped iron-oxide based nanomaterials, which were rendered water soluble by ligand displacement, were found to be non-inflammatory towards liver and kidney [26]. It suggested that trace quantities of long chain alkyl ligands on these nanoparticles are biologically safe. However, residual trace of the hydrophobic stabilizer can confer a heterogeneous mixture of hydrophobic and hydrophilic surface stabilization of SPIONs, affecting their colloidal stability and magnetic properties [17].

We report herein a simple and versatile synthetic methodology to confer aqueous solubility as well as colloidal stability to SPIONs by using phosphonate-based ligands. This was achieved by (i) a method, in which SPIONs were capped with OA, and subsequently a biphasic [16] ligand exchange reaction was performed using phosphonate-TEG-based ligands, phosphonate-TEG-Me, or phosphonate-TEG-OH; or (ii) direct conjugation, where phosphonate-TEG-Me or phosphonate-TEG-OH is conjugated to bare SPIONs. It was determined that about 30% *w*/*w* amount of phosphonate ligand was present on the conjugated SPIONs post ligand exchange reaction. TEG-based ligands were effective in imparting water-solubilizing properties to SPIONs in both protocols. SPIONs, regardless of the method employed for their translation into water-soluble nanoparticles, exhibited good dispersion compared to bare and OA terminated SPIONs. The MRI relaxivities of SPIONs containing residual OA were higher than those containing phosphonate ligands, however, these caused increased cell death compared to SPIONs prepared using direct conjugation method with no OA. We demonstrate that the nature of the ligand and the protocol employed for introducing water solubility and stability, significantly influence their overall properties. In particular, phosphonate-TEG-Me and phosphonate-TEG-OH ligands impart different colloidal and magnetic properties to SPIONs.

## 2. Results and Discussion

### 2.1. Synthesis of Ligands

**Scheme 1:**
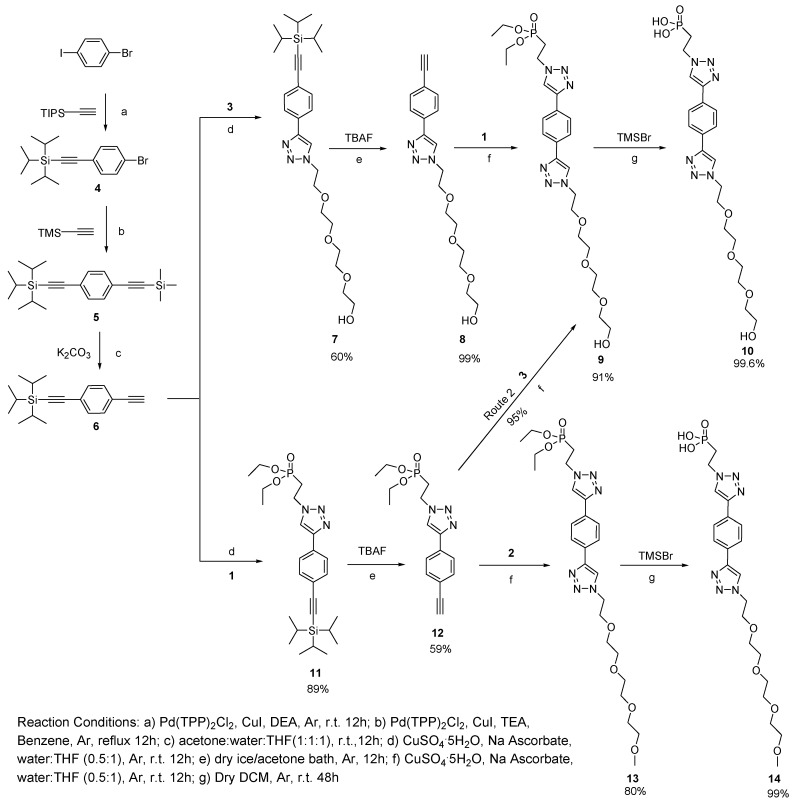
Synthetic elaboration of phosphonate-two tetraethylene glycol (TEG)-OH (**10**) and phosphonate-TEG-Me (**14**).

Two tetraethylene glycol (TEG)-based ligands with different terminal groups, (i) methyl, phosphonate-TEG-Me; and (ii) hydroxide, phosphonate-TEG-OH, were employed in this study. Their synthesis is highly versatile, and makes use of orthogonal nature of the core molecule, and robust chemistry including Sonogoshira cross-coupling and copper-catalyzed alkyne azide cycloaddition (CuAAC) “click” reactions [27,28]. The preparation of azide terminated building blocks (phosphonate-N_3_ **1**; N_3_-TEG-OMe **2**; and N_3_-TEG-OH **3**) for the click reaction, is described in Scheme S1. Synthesis of ligands was initiated from commercially available bromo-iodo-benzene with Sonogoshira coupling reactions with triisopropylacetylene (**4**, Scheme 1), followed by trimethylsilylacetylene (**5**). Selective deprotection of the latter using K_2_CO_3_, led to the formation of the core with orthogonal acetylene groups (**6**). Copper catalyzed alkyne azide coupling of **6** with HO-TEG-N_3_ (**3**), yielded **7**. Subsequent deprotection of the TIPS group in **7** in the presence of tetrabutyl ammonium fluoride (TBAF), afforded the free acetylene (**8**). It was coupled with diethyl(azido-ethyl)phosphonate (**1**) by CuAAC reaction, to give compound **9**. The dealkylation of diethylphosphonate to synthesize the desired phosphonate-TEG-OH (**10**) was achieved using excess TMSBr, a reagent commonly employed for the deprotection of alkylated phosphonates. It was necessary to use methanol to hydrolyze the silanol intermediate during the reaction with TMSBr [29].

Phosphonate ligand with methyl terminated TEG, phosphonate-TEG-Me, was prepared from **6** using a similar sequence of reactions as mentioned above: (i) CuAAC reaction with diethyl(azido-ethyl)phosphonate leading to the formation of **11**; (ii) deprotection of TiPS with TBAF (**11**); (iii) click reaction of **11** with Me-TEG-N_3_ (**2**) yielding **12**; and (iv) deprotection of phosphonate methyl groups in **13** with TMSBr, to give the desired phosphonate **14**. It should be noted that compound **14** could also be prepared from **9** using a CuAAC reaction with HO-TEG-N_3_ (**3**) (Scheme 1, Route 2).

### 2.2. Synthesis of Surface Functionalized SPIONs

The synthesis of oleic acid stabilized SPIONs (OA-SPIONs, Fe_3_O_4_), was performed by adapting the co-precipitation method [30]. The OA-SPIONs dispersed easily in organic solvents such as hexanes, ethyl acetate and chloroform, but were insoluble in an aqueous medium. To render them water-soluble, a ligand exchange reaction in a suitable solvent system is required. Previous studies have reported the use of various solvent combinations such as chloroform, DMF, ethanol, methanol, alkanes, and DMSO [23,29,31]. Our choice of solvent and its combinations, are related to the solubility of both the starting OA-SPIONs and the final ligand exchanged products. In this study, we employed chloroform to allow complete dispersion of OA-SPIONs, and a small amount of DMSO to facilitate phase-transfer to occur more readily.

The aqueous solution containing the phosphonate ligand is able to interact with polar DMSO, and allows miscibility between the solvents. The reduced volume of DMSO was critical in recuperating the final ligand-exchanged nanoparticles, SPIONs-OA/PMe, and SPIONs-OA/POH without having to perform excessive supernatant washings, which limits the loss of functionalized SPIONs. Upon ligand exchange, SPIONs were found to be dispersible by sonication in water, compared to OA-SPIONs, which were not water-dispersible.

In order to assess the optimal pH for the direct conjugation method, it is important to consider the properties of bare nanoparticles, as well as of the stabilizing phosphonate ligands. It has been reported that the isoelectric point (IEP) of bare magnetite occurs between pH 6.8 [32] to 9 [14]. It suggests that at physiological pH (pH 7.2–7.4) [14], the bare nanoparticles in aqueous suspension, have a net charge of zero, and hence have no mobility in an electric field. Below the IEP at pH between 0 and 6.8, the bare magnetite nanoparticles present a positive surface charge. Above the IEP, (generally above pH 9) they will have negative surface charge. The phosphonate ligands are reported to exhibit a pKa_1_ anywhere from 2.15 to 3, and pKa_2_ = 5 to 7.3 [33,34]. It is known that the phosphatation reaction is optimized when the surface of magnetite is positively charged, and the deprotected phosphonate ligands are negatively charged. The pH range that can afford the highest surface functionalization, thus, should lie anywhere between the pKa_1_ of phosphonic acid and IEP of bare magnetite. We chose to conjugate SPIONs at pH 4.5, a value that is a compromise between the first deprotonation of the deprotected phosphonate, and the complete lack of net charge on the surface of bare magnetite.

### 2.3. TEM, EDX, and SAED Studies of SPIONs

The oleic acid stabilized SPIONs (OA-SPIONs) dispersed readily in hexanes upon 5 s sonication, and were characterized by Transmission electron microscopy (TEM), *hkl* magnetite indexing according to d-spacing [35] calculations from the known camera constant and using Image-J software (US National Institutes of Health, Bethesda, Maryland, USA), and Energy dispersive X-ray (EDX) analysis (Figure S1). TEM analysis showed a small degree of agglomeration, and depicted a unimodal distribution centered at 7 nm. The EDX analysis correlated well with the presence of Fe and O.

**Figure 1:**
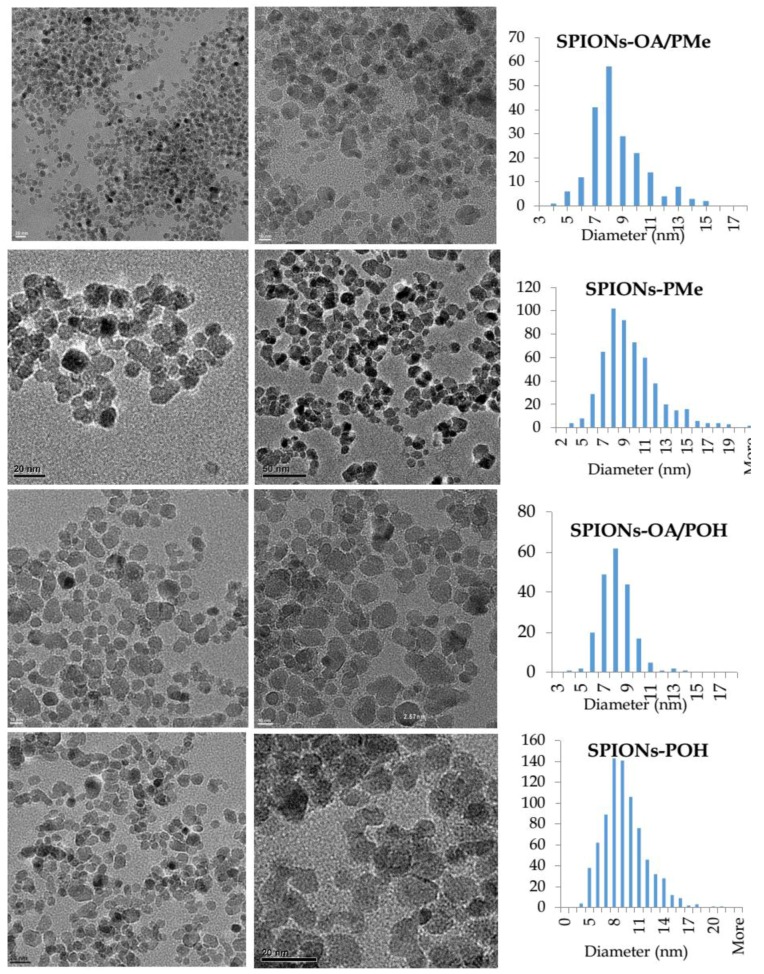
Transmission electron microscopy (TEM) images and histograms of size distribution for water-dispersible SPIONs.

The SPIONs obtained from the ligand exchange and the direct conjugation methods were dispersed in water from their dry powders, with sonication for 1 min. Figure 1 shows TEM images of SPIONs on the left-hand side, and their respective histograms of size distribution on the right, where the y-axis represents the number of nanoparticles. It should be noted that there is no difference in size between the SPIONs from ligand exchange and those obtained via the direct conjugation protocol. The SPIONs showed a unimodal size distribution with an average diameter of 8 ± 2 nm, indicative of reproducibility in the size of SPIONs between batches. It could be attributed to the systematic use of same reaction conditions to synthesize bare SPIONs. The ligand exchange and direct conjugation using phosphonate-TEG-OH did not show any significant differences in their TEM images.

Indexing of the SAED data (Figure S2) showed that all samples presented similar electron diffraction patterns, and matched the *hkl* indices of magnetite as per d-spacing measurements obtained from the known camera constant [36]. It demonstrates that the direct conjugation protocol does not cause rapid oxidation of the magnetite surface into hematite. The EDX images of all samples were very similar, with the peaks for Fe and O for iron oxide species, as well as P originating from the ligand.

### 2.4. FT-IR Studies

The Fourier transform infrared spectroscopy (FT-IR) spectral analyses showed the aliphatic C–H stretch as a large skewed shoulder between 800 and 500 cm^−1^ for OA-SPIONs, SPIONs-OA/PMe, and SPIONs-OA/POH. The C–O stretching band from TEG appeared as a broad shoulder in the region between 1200 and 850 cm^−1^ for all the samples, except OA-SPIONs (Figure S3). The C=C aromatic stretches were seen as small sharp bands between 1600 and 1450 cm^−1^. This is similar to the observations by Lamanna *et al.* pertaining to their aromatic phosphonate dendrons [33]. The P=O stretching bands appeared around 1200 cm^−1^. At higher wave numbers, the presence of aliphatic C–H stretching appeared as a broad band around 2800 cm^−1^. The key important features were the presence of aromatic C=C, and P=O stretching bands in all the samples except OA-SPIONs, indicative of the presence of the phosphonate ligands. FT-IR results provide further evidence that ligand exchange and direct conjugation, were successful in linking phosphonate ligands to the surface of SPIONs.

### 2.5. Thermogravometric Analysis (TGA)

SPIONs showed a weight decrease over increasing temperature related to the decomposition of organic components on the surface. OA-SPIONs reached a thermal weight equilibrium around 400 °C (Figure S4). One could conclude that (i) any weight loss between 100 and 400 °C, is attributed to the presence of OA in OA-SPIONs, SPIONs-OA/PMe, and SPIONs-OA/POH; and (ii) all weight loss above 400 °C to the phosphonate ligand. However, since the phosphonate ligand is also shown to have a weight decrease prior to 400 °C, exemplified by the TGA traces of SPIONs-POH and SPIONs-PMe, it is highly likely that between 100 and 400 °C, a mixture of weight loss from decomposition of both ligands occurs.

The relative percentages of OA and phosphonate ligands present on the SPIONs were calculated using the TGA data (Table S1). A few observations can be made in terms of relationships that exist between all the samples. For OA-SPIONs, the total weight loss attributed to OA is 22%, 16% for SPIONs-OA/PMe, and 23% for SPIONs-OA/POH. The data suggest that due to the nature of long alkyl chains of OA, only a maximum coverage in the vicinity of ~20% can be achieved regardless of the quantity of OA used to cap the bare SPIONs. Since the phosphonate linker also presents a weight loss prior to 400 °C, this interpretation may not entirely be quantitative. As expected, it is more likely that ligand exchange would result in a decreased presence of OA on SPIONs.

Another interesting observation is that the total weight loss percentages are quite similar for all SPIONs containing phosphonate ligands, and fall within 15% of each other. It is, therefore, possible that a maximum weight of coating is reached, considering that the surface of SPIONs have a finite surface area to allow for ligand displacement and for anchoring of the phosphonate groups. In ligand exchange, a smaller percentage of weight loss was attributed to phosphonate ligands as compared to the direct conjugation. This is expected since ligand exchange involves the displacement of long chain OA from the surface of OA-SPIONs. In contrast, the direct conjugation protocol relies on anchoring on a bare surface, leading to greater percentage of phosphonate ligand on the surface.

### 2.6. Ligand-Exchange Studies: UV-Vis

To complement TGA data, and in order to have an additional method of quantifying the amount of phosphonate-TEG-Me or phosphonate-TEG-OH present post ligand exchange, UV-Vis absorption studies were performed on the supernatant solutions. This spectroscopic method of analysis is widely used to assess the amount of ligand exchange [29,32,34], but it relies heavily on the fact that the ligand should give a UV-Vis response. In our systems, the benzyl and triazole rings confer UV-Vis transitions of the π ➔ π* and, thus, provide quantitative analysis of the amount of grafted phosphonate linker within the approximations of the Beer-Lambert Law.

The solutions of the deprotected phosphonate ligands were prepared with the same concentrations as the ones used to perform ligand exchange, and diluted accordingly to the concentrations of the supernatant, and their UV-Vis spectra were acquired. Subsequently, UV-Vis spectra of the supernatant solutions post centrifugation runs were obtained. The peak maxima, before conjugation, at 276 nm for both phosphonate-TEG-Me and phosphonate-TEG-OH represent the maximum amount of phosphonate ligand available for conjugation in solution. It can be compared with the peak maxima at 276 nm for both supernatant solutions post-conjugation, to obtain an indication of the amount of ligand that remained in solution. It should be noted that the UV-Vis spectra for the supernatant solutions for both SPIONs-OA/PMe and SPIONs-OA/POH are normalized to account for the presence of absorbance due to the remaining SPIONs in the supernatant post centrifugation. This resulted in the peak maximum for the supernatant of SPIONs-OA/PMe to become slightly blue-shifted, and the UV-Vis spectrum of the supernatant of SPIONs-OA/POH to dip below the baseline. The difference of peak maxima between the ligand and the respective normalized supernatant solutions represents the maximum amount of phosphonate linker which can conjugate onto the OA-SPIONs by displacement of OA (Table S1).

It is inferred from the data that for SPIONs-OA/PMe, a maximum percentage of ~24% of ligand exchange is achieved, whereas it is 41% for SPIONs-OA/POH (Table S2). The dip in the UV-Vis absorption spectra for normalized SPIONs-OA/POH supernatant can cause a small deviation in the calculations [29,32,34]. Despite limitations, the results from UV-Vis study are comparable to the TGA data. The phosphonate ligand weight loss by TGA attributed to SPIONs-OA/PMe was determined to be 28% and that of SPIONs-OA/POH of 35%, both values similar to the ones assessed by UV-Vis (23.4% and 41.1%), indicating that the UV-Vis normalizing method is reliable, and suggests that roughly 30% of phosphonate ligand underwent ligand exchange with the OA-SPIONs.

### 2.7. Dynamic Light Scattering

**Figure 2:**
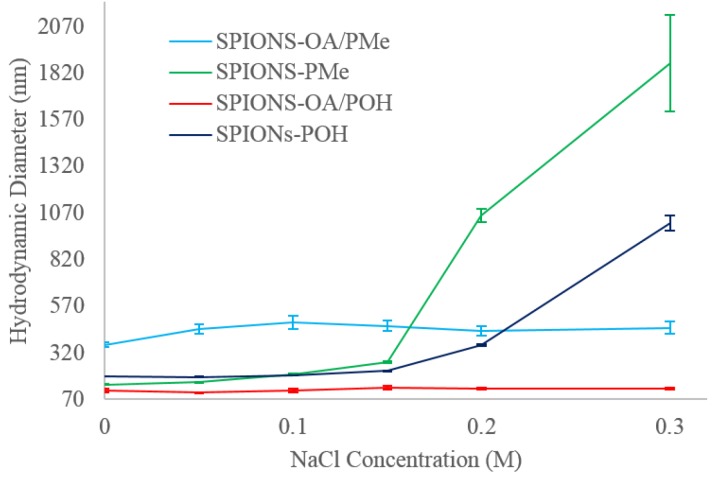
Hydrodynamic diameters measured from dynamic light scattering (DLS) at various Sodium chloride (NaCl) concentrations for all water-dispersible Super paramagnetic iron oxide nanoparticles (SPIONs).

The hydrodynamic diameters (*D*_H_) of all conjugated SPIONs at pH 7.3 in various NaCl concentrations from 0 to 0.30 M NaCl, are shown in Figure 2. SPIONs in the absence of NaCl show considerably larger diameters (from 116 to 363 nm) than those obtained from TEM (9 ± 2 nm). It has been reported that nanoparticles do not form clusters of agglomerates post stabilization, as noted by the marginal small size increase between their dynamic light scattering (DLS) *versus* TEM data [23,37]. Most other groups utilizing polymer and dendrimeric coatings have witnessed [38,39,40] a drastic size increase of as much as 200% in the hydrodynamic radii by DLS, compared to their TEM images. It was postulated to be due to incomplete surface coverage, causing agglomeration into micelle-like aggregates [41]. For our systems, in the absence of NaCl, it is likely due to the unsuccessful dispersion of agglomerated dry SPIONs prior to adding the phosphonate ligands for both conjugation methodologies that led to a large D_H_ compared to TEM measurements [42].

TEM imaging is a good tool to measure the metal core diameters for various nanoparticles, however once conjugated with an organic stabilizer, the organic coating will interact with the medium in which they are dispersed, and will present different sizes from what is seen by DLS. It is inherently a 2D imaging tool. DLS on the other hand provides a better idea of the hydrodynamic size of the dispersed nanoparticles since the technique is solution-based and, therefore, can account for the contribution of the organic coating towards the final size of the engineered nanoparticles. Since physiological conditions require a relatively high salt concentration of up to 0.15 M NaCl, it was decided to double the salt concentration to determine how the hydrodynamic diameters would be affected by the presence of high salt concentrations. In terms of overall colloidal stability, large deviation in hydrodynamic diameters are not desired, since size increases are indicative of agglomeration taking place [43]. The group of Lamanna *et al.* [33] have investigated phosphonate dendron grafted SPIONs, and noticed that the hydrodynamic sizes do not fluctuate much below a salt concentration of 0.20 M NaCl, and attributed this observation to good colloidal stability in iso-osmolar conditions.

It can be seen that ligand-exchanged SPIONs-OA/PMe and SPIONs-OA/POH possess good colloidal stabilization even at very high NaCl concentrations, as evidenced form the very marginal D_H_ variation. This suggests that OA is effective in providing steric repulsion between the nanoparticles even at elevated electrolyte concentrations, most likely due to the lack of interaction between the electrolyte and the long aliphatic chains of OA. SPIONs devoid of OA on the other hand, gradually agglomerate at elevated NaCl concentrations, indicating that the TEG moiety may interact with the electrolyte to destabilize the electrostatic repulsion of the double layer, rendering it ineffective and leading to agglomeration. Despite the colloidal instability at very high NaCl concentration for SPIONs devoid of OA, the *D*_H_ variation is still within 1 order of magnitude for all SPIONs, compared to an increase of over two orders of magnitude for high salt concentrations witnessed by Lamanna [33]. More importantly, at physiological conditions of 0.15 M, all the conjugated SPIONs possess good stability. It can be seen that SPIONs containing phosphonate-TEG-Me are larger and more influenced by the presence of electrolyte, than those containing phosphonate-TEG-OH. Thus, the end group, methyl *versus* hydroxyl, does have an effect on the overall colloidal properties of SPIONs.

### 2.8. Zeta Potential

In order to further understand the colloidal properties of conjugated SPIONs, Zeta Potential, ζ, measurements in solution at various electrolyte concentrations were performed (Figure S5). A decrease in magnitude of ζ is indicative of a decrease in electrostatic repulsion [44]. The ζ values in the absence of NaCl are all very high ranging from −55 to −27 mV, which decrease in magnitude at increasing electrolyte concentration, a trend that is comparable to previously studied SPIONs containing surface coatings such as biotin [45]. More interestingly, a previous study by Tombacz had found [46] that at pH 6.5, small carboxylic acid molecules, such as citric and gallic acid, were not large enough to stabilize magnetite nanoparticles, despite showing large ζ between −35 to −55 mV at NaCl concentration of up to 0.07 M. However, the larger carboxylic acid macromolecules such as poly(acrylic acid), poly(acrylic-co-maleic acid) and humic acid, could enhance NaCl resistance up to 0.5 M NaCl [46]. Our studies demonstrate that small TEG-phosphonate ligands are capable of conferring colloidal stability up to 0.15 M, due to a combination of both electrostatic and steric repulsions. At elevated NaCl concentrations, above 0.15 M, both of these repulsive forces are not capable of balancing the van der Waals attractive forces for the SPIONs that do not contain OA.

### 2.9. MRI Measurements

The relaxivities for SPIONs in agarose phantoms, in units of mM of Fe, and hydrodynamic diameters at pH 7.3 with no electrolyte, together with the data from other clinically available MRI probes [47,48] are presented in Table 1. In general, *r*_1_ relaxivities are low for these compounds. However, the values of *r*_2_ and *r*_2_* are very large and comparable for SPIONs-OA/PMe and SPIONs-OA/POH, indicating that they could be very effective *r*_2_ and *r*_2_* contrast agents. Taking into account confidence intervals, our *r*_2_, *r*_2_* values are well above those that are typically reported, such as those by Chapon *et al.* [49] for SPIONs of 12 nm hydrodynamic diameter, exhibiting *r*_1_ = 1.2 mM^−1^·s^−1^ , *r*_2_ = 247 mM^−1^·s^−1^, and *r*_2_/*r*_1_ = 206 measured at 7T.

### 2.10. Cytotoxicity Studies

**Figure 3:**
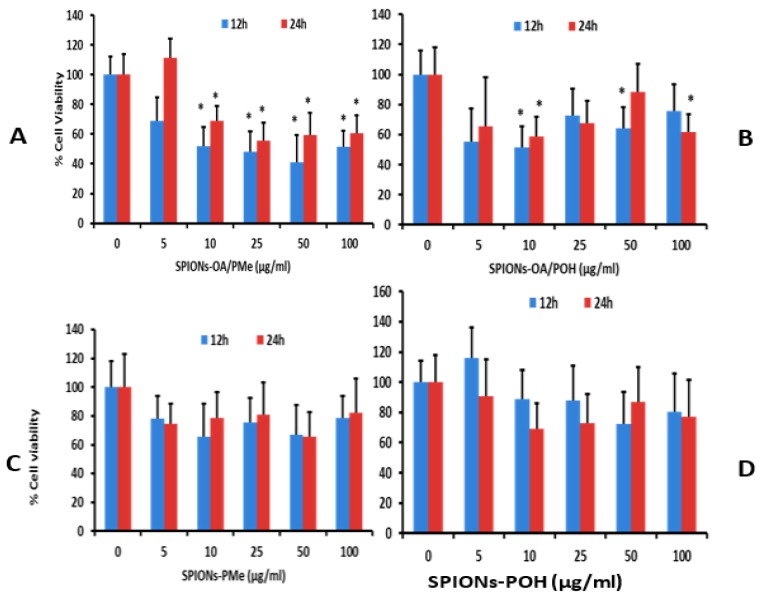
Brain endothelial cell viability after 12 and 24 h treatment with water-dispersible SPIONs at various concentrations (μg/mL): (**A**) SPIONs-OA/PMe; (**B**) SPIONs-OA/POH; (**C**) SPIONs-PMe; (**D**) SPIONs-POH.

**Figure 4:**
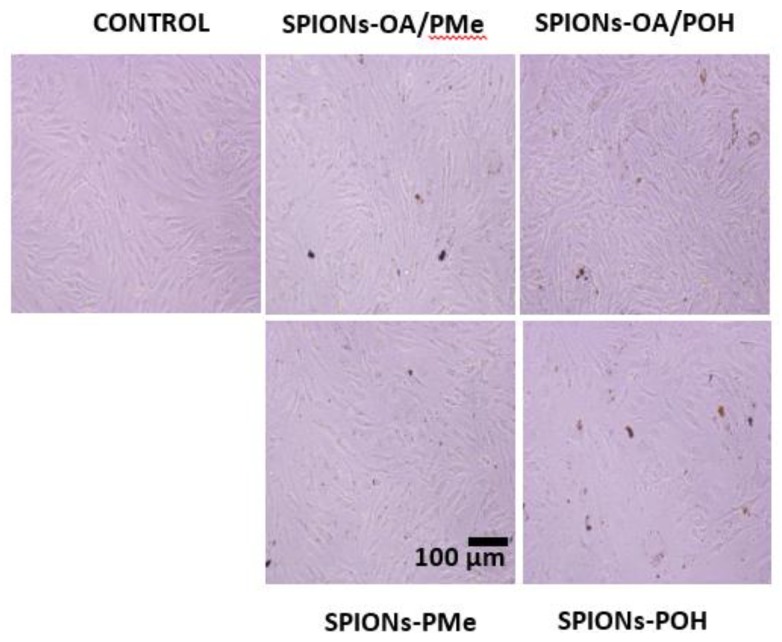
Optical images of bEnd.3 cells treated with 2.5 µg/mL of conjugated SPIONs for 24 h: Control, extreme left; SPIONs-OA/PMe, top middle; SPIONs-PMe, bottom middle; SPIONs-OA/POH, top extreme right; SPIONs-POH, bottom extreme right.

Figure 3 shows the graphs of Brain endothelial cells (bEnd.3) endothelial cell viability studies upon 12 and 24 h treatment with varying concentrations of (A) SPIONs-OA/PMe; (B) SPIONs-OA/POH; (C) SPIONs-PMe; and (D) SPIONs-POH. Figure 4 depicts the optical images of bEnd.3 cells treated with the conjugated SPIONs for 24 h, with the control cells on the left-hand side. The cellular uptake data of the conjugated SPIONs, 24 h post incubation, are presented in Table S3. The cell viability data is critically assessed in tandem: meaning that the comparisons are made relative to the method of conjugation, such that the performance of SPIONs-OA/PMe is compared to that of SPIONs-PMe. Increased cell death was witnessed 24 post-treatment for SPIONs that underwent ligand exchange protocol (Figure 3). At a concentration as low as 10 μg/mL, almost all the SPIONs containing OA caused cell death within the first 12 h. This observation was made despite the lower cellular uptake of these SPIONs, between 2% to 10% less uptake than those by direct conjugation. This observation can be rationalized by the presence of a small percentage, roughly 20%, of OA on the nanoparticles. This can lead to non-uniform coverage on the surface of SPIONs, resulting in the formation of large agglomerates for SPIONs-OA-PMe. It has been reported that long unsaturated fatty acids such as OA can cause cell death by apoptosis [50]. The presence of OA imparted both lower cellular uptake and greater cell death. Therefore larger doses of ligand exchanged SPIONs may need to be injected for the *in vivo* studies to give a good T2 and T2*-weighted MRI contrast in cellular tissue at the expense of increasing risk of toxicity. Unlike the ligand exchange process, SPIONs synthesized via direct conjugation did not result in any significant cell death despite greater percentage of uptake (100 μg/mL) over 24 h duration. From this study, it is clear that the direct conjugation of ligands to the SPIONs is the method of choice for obtaining maximum biocompatibility and cellular uptake with the least cytotoxicity.

## 3. Materials and Methods

### 3.1. Synthesis of Phosphonate-TEG-OH and Phosphonate-TEG-Me

The following compounds were synthesized by adaptation and modification of the literature procedures; Me-TEG-Mesyl [51], Me-TEG-N_3_ [52], HO-TEG-mesyl [53], HO-TEG-N_3_ [53], **1** [54], and **4**–**6** [55,56,57]. Details of the synthesis of compounds **7**–**14**, and SPIONs stabilized by oleic acid (OA) [30], are provided in the supplementary materials section.

### 3.2. Ligand Exchange: Synthesis of SPIONs-OA/PMe and SPIONs/OA-POH

For ligand exchange reaction [23,58], oleic acid stabilized SPIONs, OA-SPIONs powder (20 mg) was dissolved in 11 mL of dry and degassed chloroform:DMSO (10:1) mixture, purged with Ar at room temperature, and sonicated for 10 min prior to the addition of a degassed solution of either phosphonate-TEG-OMe (14, 100 mg, 2.0 × 10^−4^ mol) of phosphonate-TEG-OH (10, 100 mg, 2.0 × 10^−4^ mol) which were dissolved in degassed Milli-Q water (6 mL). The reaction mixtures were sonicated for 30 min at room temperature prior to being left stirring for 12 at room temperature for complete ligand exchange to take place. The flasks were subsequently opened to air and magnetic decantation was carried out, the respective murky supernatant solutions were removed by decantation and the SPIONs-OA/PMe or SPIONs-OA/POH were washed three times with anhydrous ethanol to remove excess OA and unconjugated phosphonate ligand. The final SPIONs-OA/PMe or SPIONs-OA/POH were dried using a small stream of Ar and obtained as a black powder.

### 3.3. Direct Conjugation: Synthesis of SPIONs-PMe and SPIONs-POH

The synthesis of bare SPIONs was carried out using co-precipitation method [30,59], and subsequently stabilized with the desired phosphonate ligand [33,34]. Details are provided in the supplementary materials section. A typical ligand conjugation protocol is described herein. 20 mL of degassed Mili-Q water were added to a three neck 50 mL RBF equipped with a magnetic stir bar, was degassed and purged under Ar prior to adding 50 mg of dried SPIONs powder. The flask was sonicated at room temperature under Ar for 45 min prior to the dropwise addition of a degassed aqueous (Milli-Q) solution of either phosphonate-TEG-OMe (**14**) or phosphonate-TEG-OH (**10**) (100 mg, 2.0 × 10^−^^4^ mol). The flask was allowed to sonicate for another 30 min prior to being basified to pH 4.5 using degassed NH_4_OH and left to stir for 12 h at room temperature under Ar. The resulting SPIONs-PMe (from conjugation with phosphonate-TEG-OMe) or SPIONs-POH (from conjugation with phosphonate-TEG-OH) obtained were then dispensed into centrifuge tubes and centrifuged at 10,000 rpm for 1.5 h at 4 °C, the supernatant subsequently removed, and the pellet re-dissolved in Milli-Q water and centrifuged once more. All the supernatant solutions were collected for further UV-Vis analyses.

### 3.4. MRI Relaxometry Measurements

MRI imaging was performed with a 7 Tesla scanner (Agilent, Santa Clara, CA, USA). “Gold standard” experimental conditions were put in place to ensure accuracy of relaxometry measurements. Spin echo sequences with long repetition times (*TR*) were used for all measurements. A 7 cm inner diameter volume coil (Rapid MR, Columbus, OH, USA) was used. From the MR images, 25 voxels in the center of each sample were manually selected, free from partial volume effects. Mean magnetic resonance signals (Y) were extracted as a function of echo time (*TE*) (for *T*_2_, *T*_2_* measurements) and inversion time (*TI*) (for *T*_1_ measurement), by averaging the signal over the selected voxels from each sample. Calculations of the relaxivities were done by fitting the following equations for extracting decay constants *T*_2_ and *T*_2_*: Y = A × exp(−*TE*/*T*_2_) or Y = A × exp(−*TE*/*T*_2_*), while for extracting T_1_: Y = A × (1 − 2 × B × exp(−*TI*/*T*_1_)).

### 3.5. Cell Culture and Viability Studies

Brain endothelial cells (bEnd.3) were cultured in Dulbecco’s modified Eagle’s medium 1x (DMEM medium) supplemented with 10% fetal bovine serum (FBS), and incubated at 37 °C, 5% CO_2_ in a humidified atmosphere. Viability assays were performed to determine the toxic effects of ligand conjugated SPIONs. For the toxicity assessment, cells were seeded in 96-well plates and after overnight incubation, and treated for 12 h and 24 h with various concentrations of ligand conjugated SPIONs. Cell viability was assessed using the CellTiter 96 Aqueous One Solution Assay kit according to the manufacturer instructions. Cell-Titer solution (20 µL) was added to each well 2 h before the end of each incubation duration, and the plates were incubated at 37 °C protected from light. The absorbance was recorded at 490 nm on microplate reader.

### 3.6. Cellular Uptake of SPIONs

Inductively coupled plasma optical emission spectrometry (ICP-OES) study was performed to determine the cellular uptake of the SPIONs by quantifying the iron concentration, on an Agilent Technology 5100 ICP-OES spectrometer. For the internalization study, the samples were measured three times for [Fe] and the average calculated with its standard deviation by plotting a standard curve (10–200 µg/mL, *R* = 0.9997) using λ_max_ = 238.204 nm.

## 4. Conclusions

Superparamagnetic iron oxide nanoparticles that can be easily dispersed in an aqueous medium present viable nanomaterials for a variety of biomedical applications. We have examined their synthesis using two different protocols: by ligand exchange on pre-formed oleic acid stabilized SPIONs, or by direct conjugation. The TEG based phosphonate ligands were designed and synthesized using a simple, flexible, and highly versatile synthetic methodology, which can be easily adapted to any desired platform. We demonstrate that these ligands provide good colloidal stability, magnetic and cell viability to SPIONs, and both methods of conjugation afforded spherical magnetite SPIONs that did not produce agglomerated solutions. The ligand content on the surface of SPIONs depends on the method employed for their synthesis, as a lower percentage of phosphonates was observed by ligand exchange, compared to the direct conjugation method. The cell viability of SPIONs from the direct conjugation method was higher as compared to those that underwent ligand exchange, despite the lower cellular uptake of the latter. While choosing an appropriate method for nanoparticle stability, a careful assessment of the final application needs to be evaluated in terms of a balance between the physico-chemical, magnetic, and biological profiles. The conjugation of TEG-based phosphonate-TEG-OH and phosphonate-TEG-Me ligands to SPIONs provides a useful platform in conferring colloidal stability to SPIONs, and in providing a balance of desired properties for varied applications.

## Figures and Tables

**Table 1:** Hydrodynamic diameter (*D*_H_), and relaxivities (*r*) with 10% confidence intervals for select SPIONs and water-dispersible SPIONs measured at 7T.

Nanoparticles	*D*_H_ (nm)	*r*_2_ (mM^−1^·s^−1^)	*r*_1_ (mM^−1^·s^−1^)	*r*_2_* (mM^−1^·s^−1^)	*r*_2_/*r*_1_
SPIONs-OA/PMe	363	707 (663, 751)	0.857 (0.771, 0.944)	565 (525, 606)	825 (728, 923)
SPIONs-OA/POH	116	687 (496, 878)	1.06 (0.959, 1.17)	1280 (708, 1850)	648 (458, 840)
SPIONs-PMe	149	87(81, 93)	0.159 (0.145, 0.173)	358 (181, 534)	546 (484, 608)
SPIONs-POH	193	306 (270, 343)	0.641 (0.529, 0.753)	639 (566, 712)	477 (377, 579)
Ferumoxides (Endorem^®^/Feridex^®^)	120–180	132	1.8	111.9–134.3 *	76.7
Ferucarbotran A (Resovist, SHU55A)	65	205	1.6	-	128.8

## Supplementary Materials

Details of synthesis and characterization are available online at http://www.mdpi.com/2079-4991/6/6/100/s1.
