# Supplementary material for: Fabricating Water Dispersible Superparamagnetic Iron Oxide Nanoparticles for Biomedical Applications through Ligand Exchange and Direct Conjugation
Source: Nanomaterials (Basel). 2016 May 26;6(6):100. doi: 10.3390/nano6060100 (PMC5302624; doi:10.3390/nano6060100)
Supplement: Supplementary file 1 [file nanomaterials-06-00100-s001.pdf]

# Supplementary Materials: Fabricating Water Dispersible Superparamagnetic Iron Oxide Nanoparticles for Biomedical Applications through Ligand Exchange and Direct Conjugation

**Tina Lam, Pramod K. Avti, Philippe Pouliot, Foued Maafi, Jean-Claude Tardif, Éric Rhéaume, Frédéric Lesage and Ashok Kakkar**

## Experimental

The following compounds were purchased and used as received; tetraethylene glycol, tetraethylene glycol monomethyl ether, *p*-toluene sulfonyl chloride (tosyl chloride), methane sulfonyl chloride (mesyl chloride), sodium hydroxide, lithium aluminium hydride ($LiAlH_4$), 4-dimethylaminopyridine (DMAP), triethylamine, diethyl amine, sodium azide, tetrabutylammonium iodide, sodium ascorbate (Na Ascorbate), copper(II) sulphate hexahydrate, borane-THF, bis(triphenylphosphine) palladium(II) dichloride, 1-bromo-4-iodo benzene, disodium ethylenediamine tetraacetate (EDTA), iron(II) chloride tetrahydrate ($FeCl_2{\cdot}4H_2O$), iron (III) chloride hexahydrate ($FeCl_3{\cdot}6H_2O$), and oleic acid from Sigma Aldrich (St. Louis, MO, USA), triisopropylsilyl acetylene and trimethylsilyl acetylene from Oakwoods Chemicals (Estill, SC, USA), 1-ethyl-3-(3-dimethylaminopropyl) carbodiimide from Chem Impex International (Wood Dale, IL, USA), and Iron standard # 13830 from Alfa Aesar (Haverhill, MA, USA). Polyvinylidene Fluoride (PVDF) Syringe filters of 17 mm diameter and pore size 0.45 μm were purchased from Sterlitech (Kent, WA, USA).

The solvents triethylamine, diethylamine, tetrahydrofuran, acetic acid, acetic anhydride, acetone, chloroform, dichloromethane, benzene, methanol and toluene were purchased from Fisher scientific and ACP Chemicals and used as received. Solvents were obtained from drying columns and purged under $N_2$ prior to use. Milli-Q Ultrapure water was doubly distilled by reverse osmosis though a Millipore RiOS8, followed by filtration through a Milli-Q Academic A10 filtration unit prior to use.

NMR spectral acquisitions were carried out on 300, 400, and 500 MHz Mercury (Varian) instruments and operated using VNMRJ 2.2D (Chempack 5) and VNMRJ 2.3A (Chempack 5) software, as well as on a AV 400 MHz (Bruker, Billerica, MA, USA) using a 5 mm Smart Probe. The chemical shifts in ppm are reported relative to tetramethylsilane (TMS) as an internal standard for $^1$H, and $^{13}$C {$^1$H}, and relative to $H_3PO_4$ for $^{31}$P {$^1$H} NMR spectra. Mass spectra analyses (MS-HR, ESI and APCI) were performed on an Exactive Plus Orbitrap-API (Thermo Scientific, Cambridge, MA, USA) high-resolution mass spectrometer.

Fourier transform infrared spectroscopy (FT-IR) spectra acquisitions were performed on a Spectrum TWO FT-IR with single bounce diamond ATR (Perkin Elmer, Waltham, MA, USA). Dried powdered samples of SPIONs with various stabilizing ligands (approximately 1 mg) were placed on top of the ATR diamond and data collection done in the transmission mode. The samples were swept within the IR spectrum between 500 and 4000 cm$^{-1}$.

Thermogravimetric analyses were performed on a TGA Q500 V6.7 Build 203 instrument (TA Instruments, New Castle, DE, USA). Samples for TGA were made using SPIONs with various coatings in powder form. The weight loss differential was analyzed using the TA Instrument Data Analysis Software. The powders were weighed onto a platinum pan and subjected to heating at a rate of 20 °C per min until 100 °C, kept at isothermal conditions for 2 min and subsequently ramped at 20 °C per min until 900 °C. The flow rates of air and $N_2$ gas were set at 40 mL per min and 60 mL per min respectively. The weight loss prior to the isotherm is attributed to water adsorption.

All UV-Vis spectra were acquired on a Cary 500 UV-Vis (Agilent, Lexington, MA, USA) and fluorescence spectra were obtained on a Cary Eclipse Fluorometer (Agilent) with the use of four-sided clear Quartz cuvette of 1 cm path length. A blank in Milli-Q water was performed prior to sample acquisition. All spectra were swept from 800 to 200 nm. All SPIONs obtained from the ligand exchange reaction as well as their respective ligands were subjected to UV-Vis measurements.

Carbon-coated 400 square-mesh copper grids (CF400-Cu) were purchased from Electron Microscopy Science (Hatfield, PA, USA). TEM images were obtained on a Tecnai $G^2$ F20 Cryo-STEM (FEI, Hillsboro, OR, USA) in bright-field mode at an acceleration voltage of 120 kV with an Ultrascan 4000 4 k × 4 k CCD Camera System Model 895 (Gatan, Pleasanton, CA, USA). EDX acquisitions were done using an Octane T Ultra W and Apollo XLT2 SDD and TEAM EDS Analysis System (EDAX) at a diffraction distance of 285 mm. SEAD was acquired at a diffraction distance of 340 nm and magnification of 210 k×. Samples were prepared by 30 s sonication of a hexanes aliquot of OA-SPIONs and of aqueous aliquots of bare SPIONs, SPIONs-OA/PMe, SPIONs-PMe, SPIONs-OA/POH and SPIONs-POH. A drop of the respective solution was deposited on the carbon-coated side of the Cu grid and left to stand for 30 s before being blotted and subsequently washed with water by swirling the carbon-coated side of the grid on top of water droplets and finally blotted to remove excess water. Images were processed for diameters length using the software ImageJ and preforming a calibration of the pixels based on the length of the scale bar.

Dynamic light scattering (DLS) and zeta potential (ZP) measurements were first performed with a Zeta Plus Zeta Potential Analyzer (Brookhaven Intruments, Holtsville, NY, USA) with laser set at a 90° incidence using Brookhaven Institute Particle Sizing Software and Brookhaven Zeta Potential Analyzer respectively. All the conjugated-SPIONs were dissolved in Milli-Q water adjusted to pH 7.3 as determined by pH meter (Accumet pH Meter 910, Fisher Scientific, Waltham, MA, USA) at 25 °C by diluting the conjugated-SPIONs samples to a concentration of 0.03 mg/mL adjusted for optimal scattering detection condition. The aqueous solutions were sonicated for 30 s prior to being passed through PVDF membrane filters of 0.45 μm pore size and added to polystyrene cuvettes carefully in order to avoid air bubble formation. The conjugated SPIONs in aqueous conditions were subjected to varying salt concentrations ranging from 0 M to 0.30 M NaCl using a stock solution of NaCl (aq), their hydrodynamic sizes and electro-kinetic potential sampled by DLS and Zeta Plus Zeta Potential Analyzer (ZP). DLS acquisition lasted 2.5 min and averaged over 10 measurements, while ZP measurements were averaged over 10 measurements.

The following compounds were synthesized by adaptation and modification of the literature procedures: diethyl(azido-ethyl)phosphonate, **1** [1], Me-TEG-Mesyl [2], Me-TEG-$N_3$ [3], HO-TEG-mesyl [4], HO-TEG-$N_3$ [4], and **4**–**6** (Scheme 1) [5–7]. Synthetic details for compounds **1**–**3** are provided in Scheme S1 below. Briefly, diethyl(azido-ethyl)phosphonate (**1**) was synthesized in quantitative yield by dissolving commercially available 2-bromoethylphosphonate in ethanol and reacting it with sodium azide, used in excess, under argon for 12 h. Azide terminated tetraethylene glycol ligand **2** was prepared by dissloving Me-TEG-OH in THF, followed by the addition of mesyl chloride and triethylamine, and the mixture was stirred under argon at 0 °C for 2 h. The Me-TEG-Mesyl was subsequently reacted with sodium azide in ethanol at 77 °C for 12 h. Azide terminated tetraethylene glycol ligand **3** was prepared by following a similar procedure as described here for **2**.

**Synthesis of 7** (**Scheme 1**)**:** In a 50 mL round bottom flask (RBF) purged with argon, **6** (1.02 g, 3.6 mmol), HO-TEG-$N_3$ (0.79 g, 3.6 mmol), and sodium ascorbate (286 mg, 1.44 mmol) were dissolved in 4 mL THF, and stirred at room temperature for 5 min, before the dropwise addition of a 0.8 mL aqueous solution of $CuSO_4{\cdot}5H_2O$ (180 mg, 0.72 mmol). The reaction mixture changed from yellow to brown upon addition of $CuSO_4$ solution, and then back to yellow within the course of 3 min. The mixture was stirred for 12 h at room temperature, and subsequently rotovaped to remove THF, and the remaining orange oil was dissolved in dichloromethane (DCM, 10 mL) and extracted against water (3 × 5 mL). The organic phase was then dried using anhydrous $MgSO_4$, filtered and concentrated in vacuo to afford the brownish oil, which was subjected to silica column chromatography first in pure DCM, and then the polarity was increased to obtain **7** as a colourless oil (1.09 g, 60% yield). $^1$H NMR ($CDCl_3$, 400 MHz): (ppm) δ 1.13 (s, 21H, –Si–C$\underline{H}$–and–Si–H-C$\underline{H_3}$), 3.57–3.63 (m, 10H, –C$\underline{H_2}$–C$\underline{H_2}$–O–), 3.69 (t, 2H, –O–C$\underline{H_2}$–$CH_2$–, $J_{H-H}$ = 4.4 Hz,), 3.90 (t, 2H, –O–C$\underline{H_2}$–$CH_2$-, $J_{H-H}$ = 4.4 Hz), 4.59 (t, 2H, –N–C$\underline{H_2}$–$CH_2$–, $J_{H-H}$ = 4.4 Hz), 7.52 (d, 2H, Ar, $J_{H-H}$ = 8 Hz,), 7.79 (d, 2H, Ar, $J_{H-H}$ = 8 Hz,), 8.04 (s, 1H, triazole). $^{13}$C {$^1$H} NMR ($CDCl_3$, 75 MHz): (ppm) δ 11.2, 18.64, 50.31, 61.48, 69.41, 70.07, 70.25, 70.39, 70.43, 72.47, 91.34, 106.87, 121.54, 122.96, 125.32, 130.57, 132.46, 146.87.

Measured HR-MS (APCI + ve Mode) m/z = 502.3120, calculated m/z = 502.3096 for $C_{27}H_{44}N_3O_4Si$ ([M + H]$^+$).

**Synthesis of 8:** In a 50 mL RBF purged under Ar, **7** (650 mg, 1.30 mmol) was dissolved in 3 mL of dry THF, and stirred in dry ice/acetone bath for 5 min before the dropwise addition of TBAF (1.56 mL, 1M TBAF solution in THF). This mixture was stirred for 12 h in a dry ice/acetone bath under Ar. The mixture was then rotovaped to remove THF, and the remaining brown oil was dissolved with EtOAc (10 mL). The organic phase was then dried using anhydrous $MgSO_4$ filtered and concentrated in vacuo to afford the brownish oil, which was subjected to silica column chromatography first in pure DCM, and then the polarity was increased to 1.5% MeOH:DCM to remove organic impurity, and then increased to 2.5% MeOH:DCM to obtain **8** as a colorless oil (445 mg, 99.6% yield) $^1$H NMR ($CDCl_3$, 300 MHz): (ppm) δ 2.74 (s, 1H, –OH), 3.12 (s, 1H, Ph–≡–H), 3.52–3.60 (m, 10H, –$CH_2$–$CH_2$–O–), 3.67 (t, 2H, –O–$CH_2$–$CH_2$–, $J_{H-H}$ = 4.8 Hz), 3.87 (t, J = 4.4 Hz, 2H, –O–$CH_2$–$CH_2$–), 4.59 (t, 2H, –N–$CH_2$–$CH_2$–, $J_{H-H}$ = 4.4 Hz), 7.52 (d, 2H, Ar, $J_{H-H}$ = 8 Hz, 7.79 (d, 4H, Ar, $J_{H-H}$ = 8 Hz), 8.04 (s, 1H, triazole). $^{13}$C {$^1$H} NMR ($CDCl_3$, 75 MHz): (ppm) δ 50.36, 61.58, 69.46, 70.15, 70.33, 70.45, 70.48, 72.42, 77.91, 83.49, 121.49, 121.59, 125.46, 131.19, 132.58, 146.58. Measured HR-MS (APCI + ve Mode) m/z = 346.1770, calculated m/z = 346.1761 for $C_{18}H_{24}N_3O_4$ ([M + H]$^+$).

**Synthesis of 9:** In a 50 mL RBF purged under Ar, **1** (400 mg, 1.16 mmol), **8** (240 mg, 1.16 mmol), and Na-Ascorbate (92 mg, 0.46 mmol) were dissolved in 3 mL degassed THF, and stirred at room temperature for 5 min before the dropwise addition of a 0.4 mL aqueous solution of $CuSO_4{\cdot}5H_2O$ (58 mg, 0.23 mmol). The reaction mixture changed colour from yellow to brown upon addition of the $CuSO_4$ solution, and then back to yellow within the course of 3 min. This mixture was stirred for 12 h, rotovaped to remove THF, and the remaining orange oil was dissolved with DCM (75 mL) and extracted against water (3 × 15 mL). The organic phase was then dried using anhydrous $MgSO_4$, filtered and concentrated in vacuo to afford the brownish oil, which was subjected to silica column chromatography first in pure DCM, and then the polarity was increased to 1% MeOH:DCM to obtain **9** as a yellow oil (583 g, 91% yield).

**Synthesis of Phosphonate-TEG-OH** (**10**)**:** In a 10 mL RBF purged under Ar, **9** (583 mg, 1.06 mmol) was dissolved in 3 mL dry chloroform, and stirred at room temperature for 5 min before the dropwise addition of TMSBr (0.39 mL, 2.85 mmol). This mixture was stirred for 48 h at room temperature under Ar. The yellow solution was then rotovaped to remove chloroform, dissolved in MeOH and rotovaped again to remove excess TMSBr. The yellow oil was dissolved and re-rotovaped 3 times in MeOH (15 mL each time) to afford phosphonate-TEG-OH (**10**) as a yellow oil (445 mg, 99% yield). $^1$H NMR (MeOD, 500 MHz): (ppm) δ 2.50–2.59 (m, 2H, –N–$CH_2$–$CH_2$–P–), 3.55–3.63 (m, 10H, (–$CH_2$–$CH_2$–O–), 3.66–3.68 (m, 2H, –O–$CH_2$–$CH_2$–), 4.01 (t, 2H, –O–$CH_2$–$CH_2$–, $J_{H-H}$ = 5 Hz), 4.77–4.82 (m, 4H, P–$CH_2$– and –N–$CH_2$–$CH_2$–O–), 4.58 (t, 2H, –N–$CH_2$–$CH_2$–, $J_{H-H}$ = 4.5 Hz), 7.96–8.00 (m, 2H, Ar), 8.75 (br s, 1H, triazole), 8.81 (br s, 1H, triazole). $^{13}$C {$^1$H} NMR ($CDCl_3$, 125 MHz): (ppm) δ 26.99, 28.09, 46.43, 52.15, 60.76, 68.16, 69.93, 70.01, 70.04, 70.08, 72.16, 123.65, 124.71, 126.65, 126.82, 127.35, 129.03, 143.87, 144.61. $^{31}$P {$^1$H} NMR ($CDCl_3$, 80 MHz): (ppm) δ 22.50. Measured HR–MS (ESI + ve Mode) m/z = 519.1740, calculated m/z = 519.1728 for $C_{20}H_{29}N_6O_7P$ ([M + Na]$^+$).

**Synthesis of 11:** In a round bottom flask (RBF) purged under Ar, **6** (46 mg, 0.16 mmol), diethyl(2-azidoethyl) phosphonate (**1**) (34 mg, 0.16 mmol) and sodium ascorbate (Na Ascorbate) (13 mg, 0.065 mmol) were dissolved in 2 mL of degassed THF and stirred at room temperature for 5 min under Ar before dropwise addition of a 0.4 mL aqueous solution of $CuSO_4{\cdot}5H_2O$. The solution changed colour from light yellow to brown upon injection of the Cu solution, to bright yellow and then finally to blue. The blue solution was left to stir at room temperature for 12 h under Ar. Subsequently, THF was evaporated and the crude mixture was dissolved in DCM (50 mL) and extracted against water (10 mL). The organic phase was dried using $MgSO_4$, filtered and concentrated to yield a light brown oil, which was purified on silica column chromatography firstly using pure DCM, then increasing the polarity to 0.5% MeOH:DCM, then to 1% MeOH:DCM to afford **11** as a semi–yellow crystalline oil (70 mg, 89% yield). $^1$H NMR ($CDCl_3$, 200 MHz): (ppm) δ 1.14 (s, 21H, triisopropyl H), 1.29 (t, 6H, –$CH_3$, $J_{H-H}$ = 7 Hz), 2.39–2.52 (m, 2H, –N–$CH_2$–$CH_2$–P), 4.01–4.16 (m, 4H, –$CH_2$–$CH_3$), 4.65–4.71 (m, 2H, –N–$CH_2$–$CH_2$–), 7.53 (d, 2H, $H_{Ar}$, $J_{H-H}$ = 8.2 Hz), 7.77 (d, 2H, $H_{Ar}$, $J_{H-H}$ = 8.2 Hz), 7.88 (s, 1H, triazole).

$^{13}$C {$^{1}$H} NMR ($CDCl_3$, 125 MHz): (ppm) δ 11.31, 12.17, 16.33, 16.37, 18.66, 18.76, 26.68, 27.80, 44.66, 62.15, 62.20, 91.56, 106.79, 120.41, 123.29, 125.10, 125.35, 130.19, 132.55, and 147.80. $^{31}$P {$^{1}$H} NMR ($CDCl_3$, 80 MHz): (ppm) δ 25.59. Measured HR-MS (ESI + ve Mode) m/z = 512.2469, calculated m/z = 512.2469 for $C_{25}H_{40}N_3O_3PSiNa$ ([M + Na]$^+$).

**Synthesis of 12:** In a 10 mL RBF, **11** (30 mg, 0.061 mmol) was dissolved and stirred in 2 mL of dry THF and cooled in an acetone/dry ice bath and purged under $N_2$ for 5 min prior to dropwise addition of tetrabutylammonium fluride (TBAF) (0.070 mL, 7.35 × $10^{-5}$ mol, 1M solution in THF). The reaction was stirred for 12 h and left to warm up to room temperature. The resulting yellow solution was evaporated to remove THF, dissolved in EtOAc (20 mL), and extracted against water (3 × 40 mL). The organic phase was dried with $MgSO_4$, filtered and concentrated to yield a brown oil which was purified on silica column first using pure DCM and the polarity increased to 0.5% MeOH:DCM to remove the TIPS-OH impurity, then the polarity increased to 1% MeOH:DCM to recuperate **12** as a beige powder upon concentration (12 mg, 59% yield). $^{1}$H NMR ($CDCl_3$, 500 MHz): (ppm) δ 1.29 (t, 6H, –C$\underline{H_3}$, $J_{H–H}$ = 7 Hz), 2.43–2.50 (m, 2H, –N–C$\underline{H_2}$–$CH_2$–P), 3.13 (s, 1H, acetylene H), 4.06–4.12 (m, 4H, –C$\underline{H_2}$–$CH_3$), 4.65–4.71 (m, 2H, –N–C$\underline{H_2}$–$CH_2$–), 7.54 (d, 2H, $H_{Ar}$, $J_{H–H}$ = 8 Hz), 7.78 (d, 2H, $H_{Ar}$, $J_{H–H}$ = 8 Hz), 7.88 (s, 1H, triazole). $^{13}$C {$^{1}$H} NMR ($CDCl_3$, 125 MHz): (ppm) δ 16.34, 16.38, 26.66, 27.79, 44.68, 62.18, 62.22, 77.96, 83.40, 120.50, 121.82, 125.49, 130.77, 132.66, and 147.01. $^{31}$P {$^{1}$H} NMR ($CDCl_3$, 80 MHz): (ppm) δ 25.55. Measured HR-MS (ESI + ve Mode) m/z = 356.1142, calculated m/z = 356.1134 for $C_{16}H_{20}N_3O_3PNa$ ([M + Na]$^+$).

**Synthesis of 13:** In 1 mL of degassed THF purged under Ar were dissolved **MeO-TEG-N$_3$** (**2**, 144 mg, 6.18 × $10^{-4}$ mol), **12** (206 mg, 6.18 × $10^{-4}$ mol), and Na-Ascorbate (49 mg, 2.47 × $10^{-4}$ mol) for 3 min prior to addition of a 0.5 mL aqueous solution of $CuSO_4·5H_2O$ (30.9 mg, 1.24 × $10^{-4}$ mol) and left to stir for 12 h at room temperature under Ar. The resulting yellow mixture was subsequently evaporated to remove THF and then extracted with DCM (3 × 20 mL) against water (30 mL). The organic phases were combined and dried with MgSO4, filtered and concentrated to obtain **13** as a pale yellow oil (280 mg, 80% yield). $^{1}$H NMR ($CDCl_3$, 400 MHz): (ppm) δ 1.26 (t, 6H, –C$\underline{H_3}$, $J_{H–H}$ = 6.8 Hz), 2.40–2.48 (m, 2H, –N–C$\underline{H_2}$–$CH_2$–P), 3.29 (s, 3H, –O–C$\underline{H_3}$), 3.45–3.47 (m, 2H, –C$\underline{H_2}$–O–$CH_3$), 3.54–3.60 (m, 10H, –C$\underline{H_2}$–C$\underline{H_2}$–O–), 3.88 (t, 2H, –N–$CH_2$–C$\underline{H_2}$–, $J_{H–H}$ = 5.2 Hz), 4.02–4.09 (m, 4H, –C$\underline{H_2}$–$CH_3$), 4.56 (t, 2H, –N–C$\underline{H_2}$–$CH_2$, $J_{H–H}$ = 5.2 Hz), 4.62–4.69 (m, 2H, –P–C$\underline{H_2}$–$CH_2$–), 7.83–7.90 (m, 5H, Ar and triazole), 8.03 (s, 1H, triazole). $^{13}$C {$^{1}$H} NMR ($CDCl_3$, 125 MHz): (ppm) δ 16.34, 16.38, 26.68, 27.81, 44.63, 50.39, 58.97, 62.16, 62.21, 69.50, 70.48, 70.55, 70.57, 70.59, 70.86, 120.171, 121.128, 126.06, 126.11, 130.01, 130.66, 147.20, 147.44. $^{31}$P ($^{1}$H) NMR ($CDCl_3$, 162 MHz): (ppm) δ 25.64. Measured HR-MS (ESI + ve Mode) m/z = 589.2525, calculated m/z = 589.2510 for $C_{25}H_{39}N_6O_7PNa$ ([M + Na]$^+$).

**Synthesis of Phosphonate-TEG-Me (14):** In 2 mL of dry chloroform, **13** (280 mg, 4.94 × $10^{-4}$ mol) was dissolved and stirred at room temperature under Ar prior to dropwise addition of trimethylsilyl bromide (TMSBr) (0.18 mL, 1.33 mmol). The mixture was left to stir over 48 h prior to quenching with MeOH, and concentrating the mixture into a yellow viscous oil. Upon dissolving the oil with DCM (5 mL) and MeOH (10 mL) and rotovaping, redissolving and evaporating a total of three times, the product eventually solidifies into an off-white lilac solid after being left *in vacuo* to yield phosphonate-TEG-Me (**14**) (200 mg, 79% yield). $^{1}$H NMR (MeOD, 400 MHz): (ppm) δ 2.43–2.52 (m, 2H, –N–C$\underline{H_2}$–$CH_2$–P), 3.27 (s, 3H, –O–C$\underline{H_3}$), 3.45 (s, 2H, –C$\underline{H_2}$–O–$CH_3$), 3.52–3.67 (m, 10H, –C$\underline{H_2}$–C$\underline{H_2}$–O–), 3.98 (t, 2H, –N–$CH_2$–C$\underline{H_2}$–, $J_{H–H}$ = 4.8 Hz), 4.71 (t, 2H, –N–C$\underline{H_2}$–$CH_2$–, $J_{H–H}$ = 4.8 Hz), 7.95 (s, 4H, Ar), 8.59 (s, 1H, triazole), 8.64 (s, 1H, triazole). $^{13}$C {$^{1}$H} NMR ($CDCl_3$, 125MHz): (ppm) δ 22.79, 27.36, 28.46, 45.48, 50.94, 57.60, 68.62, 69.86, 70.07, 70.05, 71.47, 122.05, 123.01, 126.08, 126.15, 129.17, 129.83, 145.64, 146.09. $^{31}$P ($^{1}$H) NMR ($CDCl_3$, 162 MHz): (ppm) δ 22.68. Measured HR–MS (ESI + ve Mode) m/z = 533.1892, calculated m/z = 533.1884 for $C_{21}H_{31}N_6O_7PNa$ ([M + Na]$^+$).

*Route 2 for the Synthesis of* ***9***: In a 10 mL RBF purged under Ar, **12** (228 mg, 6.8 × $10^{-4}$ mol), OH–TEG–$N_3$ (**3**, 150 mg, 6.8 × $10^{-4}$ mol), and Na Ascorbate (54 mg, 2.7 × $10^{-4}$ mol) were dissolved in 3 mL degassed THF, and stirred at room temperature for 5 min before the dropwise addition of a 0.4 mL aqueous solution of $CuSO_4·5H_2O$ (34 mg, 1.4 × $10^{-4}$ mol). The reaction mixture changed from yellow to brown upon addition of the $CuSO_4$ solution and then back to yellow, afterwards. This mixture was stirred for 12 h at room temperature under Ar. The mixture was then rotovaped to remove THF, and

the remaining yellow oil was dissolved with DCM (75 mL) and extracted against water (3 × 15 mL). The organic phase was then dried using anhydrous $MgSO_4$, filtered and concentrated in vacuo to afford **9** as yellow oil (360 mg, 95% yield). $^1H$ NMR ($CDCl_3$, 300 MHz): (ppm) δ 1.28 (t, 6H, –C$\underline{H_3}$, $J_{H-H}$ = 7.2 Hz), 2.42–2.51 (m, 2H, –N–C$\underline{H_2}$–$CH_2$–P), 3.51–3.61 (m, 10H, –C$\underline{H_2}$–C$\underline{H_2}$–O–), 3.67 (t, 2H, –O–C$\underline{H_2}$–$CH_2$–, $J_{H-H}$ = 4.4 Hz,), 3.88 (t, 2H, –O–C$\underline{H_2}$–$CH_2$–, $J_{H-H}$ = 4.8 Hz), 4.02–4.11 (m, 4H, –C$\underline{H_2}$–$CH_3$), 4.58 (t, 2H, –N–$CH_2$–C$\underline{H_2}$–, $J_{H-H}$ = 4.5 Hz), 4.62–4.68 (m, 2H, –P–C$\underline{H_2}$–$CH_2$–), 7.84–7.88 (m, 5H, Ar and triazole), 8.05 (s, H, triazole). $^{13}C$ {$^1H$} NMR ($CDCl_3$, 75 MHz): (ppm) δ 16.31, 16.39, 26.27, 28.13, 44.60, 50.35, 61.57, 62.13, 69.51, 70.19, 70.36, 70.51, 72.44, 120.27, 121.28, 126.06, 126.10, 130.00, 130.64, 147.40. $^{31}P$ {$^1H$} NMR ($CDCl_3$, 80 MHz): (ppm) δ 25.66. Measured HR-MS (APCI + ve Mode) m/z = 553.2523, calculated m/z = 553.2534 for $C_{24}H_{38}N_6O_7P$ ($[M + H]^+$).

**Synthesis of OA-SPIONs:** In a three neck 100 mL RBF flask equipped with a water condenser and purged with Ar, 20 mL of Mili-Q water were degassed before the addition of $FeCl_2·4H_2O$ (290 mg, 1.44 mmol), and the solution was stirred at room temperature under Ar for 5 min. $FeCl_3·6H_2O$ (790 mg, 2.88 mmol) was then added, and allowed to stir for 5 min at room temperature, before immersing the flask in an oil bath at 85 °C for 10 min to yield a clear orange solution. A solution of $NH_4OH$ (aq) (28% by volume, 0.89 mL) was then injected rapidly into the reaction mixture to give a black solution, and allowed to stir under Ar at 85 °C for 30 min, prior to the dropwise addition of oleic acid (OA) (1.00 mL, 2.54 mmol). The mixture was stirred at 85 °C for another 30 min. The OA-SPIONS quickly coalesced onto the magnetic stir bar after 10 min post injection. The supernatant was removed via a syringe, and the reaction flask was washed 3 times with degassed Milli-Q water (3 × 20 mL), and 3 times with absolute ethanol (3 × 20 mL). Each washing cycle was removed via syringe. The final washed and decanted OA-SPIONs were heated to 50 °C to remove excess solvent in vacuo and recuperated as a black powder.

**Direct Conjugation: Synthesis of SPIONs-PMe and SPIONs-POH:** The synthesis of bare SPIONs was carried out using co-precipitation method, and subsequently stabilized with the desired phosphonate ligand. The synthesis of bare SPIONs is described briefly herein. In a three neck 100 mL RBF flask purged under Ar, 20 mL of Mili-Q water was degassed and purged under Ar before the addition of $FeCl_2·4H_2O$ (290 mg, 1.44 mmol) which was stirred at room temperature for 5 min. $FeCl_3·6H_2O$ (780 mg, 2.88 mmol) was then added to the previous solution and allowed to stir for 5 min at room temperature before immersing the flask in an oil bath at 85 °C for 10 min resulting in a clear orange solution. A solution of $NH_4OH$ (aq) (28% by volume, 0.89 mL) was then injected rapidly into the reaction mixture to give a black solution and allowed to stir under Ar at 85 °C for 1 h. Magnetic decantation was performed to remove the supernatant via a syringe, and the black colloidal suspension was washed 3 times with degassed Milli-Q water (3 × 20 mL) and 3 times with absolute ethanol (3 × 20 mL) and decanted each washing cycle before being heated to 50 °C to remove excess solvent under vacuum. The bare SPIONs were then stored as a black powder in the glovebox for subsequent functionalization with a given phosphonate ligands.

*Cell Culture and Viability Studies*

All SPIONs were dispersed in sterile deionized water and bath sonicated continuously for 40 min with a final output power of 150 Watts. During the entire sonication cycle, ice was used to cool the water bath to decrease rising temperature. Brain endothelial cells (bEnd.3) (Cat No.: CRL-2299, ATCC) were cultured in Dulbecco's modified Eagle's medium 1× (DMEM medium, Cat No.: 319-005-CL, Wisent Inc., Saint-Jean-Baptiste, QC, Canada) supplemented with 10% fetal bovine serum (FBS) (Gibco/Invitrogen, Grand Island, NY, USA) and incubated at 37 °C, 5% $CO_2$ in a humidified atmosphere. Unless otherwise stated, all experiments were performed by seeding the cells at a density of $3 \times 10^4$ cells/cm$^2$. Viability assays were performed on the endothelial cells to determine the toxic effects of ligand conjugated SPIONs. For the toxicity assessment, cells were seeded in 96-well plates and after overnight incubation, cells were treated for 12 h and 24 h with or without various concentrations of ligand conjugated SPIONs. The concentrations for each SPION samples varied from 5 to 100 μg/mL. Interference from the ligand conjugated SPIONs was assessed by addition of the assay reagents with the above concentration of SPIONs but without cells. Cell viability was assessed

using the CellTiter 96 Aqueous One Solution Assay kit (G3580, Promega, Madison, WI, USA) according to the manufacturer instructions. Cell-Titer solution (20 μL) was added to each well 2 h before the end of each incubation duration and the plates were incubated at 37 °C protected from light. The absorbance was recorded at 490 nm on microplate reader (BioTek Synergy 2, Winooski, VT, USA).

*Cellular Uptake of SPIONs*

Inductively coupled plasma optical emission spectrometry (ICP-OES) study was performed to determine the cellular uptake of the SPIONs by quantifying the iron concentration. For the cellular uptake of SPIONs, bEnd.3 cells were cultured in DMEM medium supplemented with 10% FBS and incubated at 37 °C, 5% $CO_2$ in a humidified atmosphere. The samples for ICP-OES were prepared as follows. Approximately $1 \times 10^6$ cells were treated with various SPIONs for 12 h. At the end of the incubation time, cells were thoroughly washed with PBS to remove unbound and excess SPIONs in the cell media. The cells were then trypsinized and then centrifuged at 130× *g* for 5 min to collect the cell pellet. The cell pellets were digested with hot $HNO_3$ for 10 min to release uptaken SPIONs, and made into a final acid solution to 5% *v*/*v* with Nano-Q water. This solution was filtered through 0.2 μm filter before being used for the ICP-OES analysis. The ICP-OES was performed using an Agilent Technology 5100 ICP-OES spectrometer (Santa Clara, CA, USA) using the standard curve prepared from iron standards. For the internalization study, the samples were measured 3 times for [Fe] and the average calculated with its standard deviation by plotting a standard curve (10 to 200 μg/mL, R = 0.9997) using $\lambda_{max}$ = 238.204 nm.

*MRI Relaxometry Measurements*

For measuring MRI properties, 6 dilutions of the SPION samples in 1% agarose were prepared. To help with solubility, SPIONs were dispersed in 100% ethanol with 0.25 M NaOH, heated to 55 °C and sonicated for 10 min. SPION stocks were then put in a BSA solution, such that the resulting content was 75% water, 25% ethanol, 0.06 M NaOH and 1% BSA. Care was taken to keep these proportions constant through the dilutions and that no precipitates of the SPIONs were noticeable. Six two-fold dilutions starting from a concentration of 1 mM Fe were thus generated in 1% agarose wt/vol, 75% water, 25% ethanol, 0.06 M NaOH and 1% BSA, prepared in 0.5 mL vials and stored at 4 °C until imaging 3 days later. Imaging time was about 32 h.

MRI imaging was performed with a 7 Tesla scanner (Agilent, Santa Clara, CA, USA). "Gold standard" experimental conditions were put in place to ensure accuracy of relaxometry measurements. Spin echo sequences with long repetition times (*TR*) were used for all measurements. A 7 cm inner diameter volume coil (Rapid MR, Germany) was used. From the MR images, 25 voxels in the center of each sample were manually selected, free from partial volume effects. Mean magnetic resonance signals (Y) were extracted as a function of echo time (*TE*) (for $T_2$, $T_2$* measurements) and inversion time (*TI*) (for $T_1$ measurement), by averaging the signal over the selected voxels from each sample. Calculations of the relaxivities were done by fitting the following equations for extracting decay constants $T_2$ and $T_2$*: $Y = A \times \exp(-TE/T_2)$ or $Y = A \times \exp(-TE/T_2^*)$, while for extracting $T_1$: $Y = A \times (1 - 2 \times B \times \exp(-TI/T_1))$. The repetition time for the evaluation of $T_1$ and $T_2$ was *TR* = 20 sec while it was *TR* = 5 s for $T_2$* measures. The echo times (*TE*) for $T_2$ measurements were: *TE* = 5.9, 6.8, 8, 10, 12, 15, 20, 25, 35, 45, 60, 75, 90, 120, 240, 400, 640, 1000 ms, while for $T_2$* they were *TE* = 2.1, 3, 4, 5, 7, 10, 15, 20, 25, 30 ms, and for *T1*, *TE* = 6 ms. For *T1* measurements, the inversion times were *TI* = 0.0075, 0.01, 0.03, 0.05, 0.07, 0.1, 0.3, 0.5, 0.7, 0.9, 1.1, 1.4, 1.7, 2.1, 2.5, 3.0, 4.0, 6.0 s. Finally, for each SPIONs sample, the $r_2$, $r_2$* and $r_1$ relaxivities (where $r_i = 1/T_i$) were calculated by least-square fitting the $T_2$, $T_2$* and $T_1$ previously calculated for each sample against the SPION sample Fe concentration.

*Statistical Analysis*

For statistical analyses on the relaxivities, the 90% confidence interval for the least-square fit was given. For cell viability analyses, all data were expressed as mean ± standard deviation (SD) ($n$ = 3,

independent replicates). The statistical significance of difference between treatment groups was analyzed using student's *t*-test with $p < 0.05$ considered significantly different.

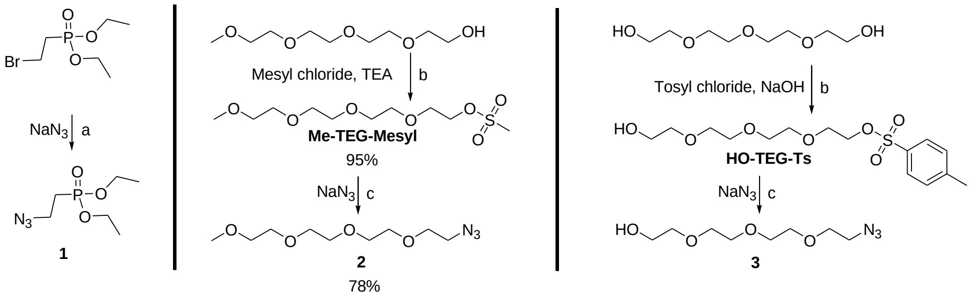


Reaction Conditions: a) EtOH, Ar, reflux 12h; b) THF, Ar, 0°C. 2h c) EtOH, Ar, 77°C, 12h;

**Scheme S1.** Synthesis of azido-terminated TEG.

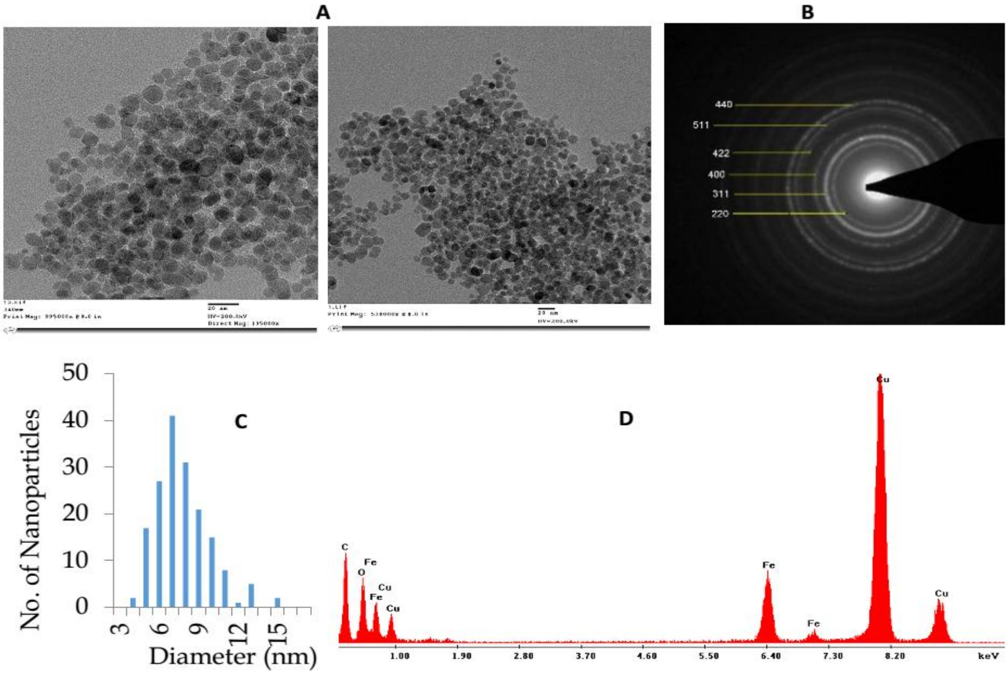


**Figure S1.** (**A**) Transmission electron microscopy (TEM); (**B**) Indexed selected area electron diffraction SAED; (**C**) histogram of TEM size distribution; (**D**) Energy dispersive X-ray (EDX) analyses of OA-SPIONs.

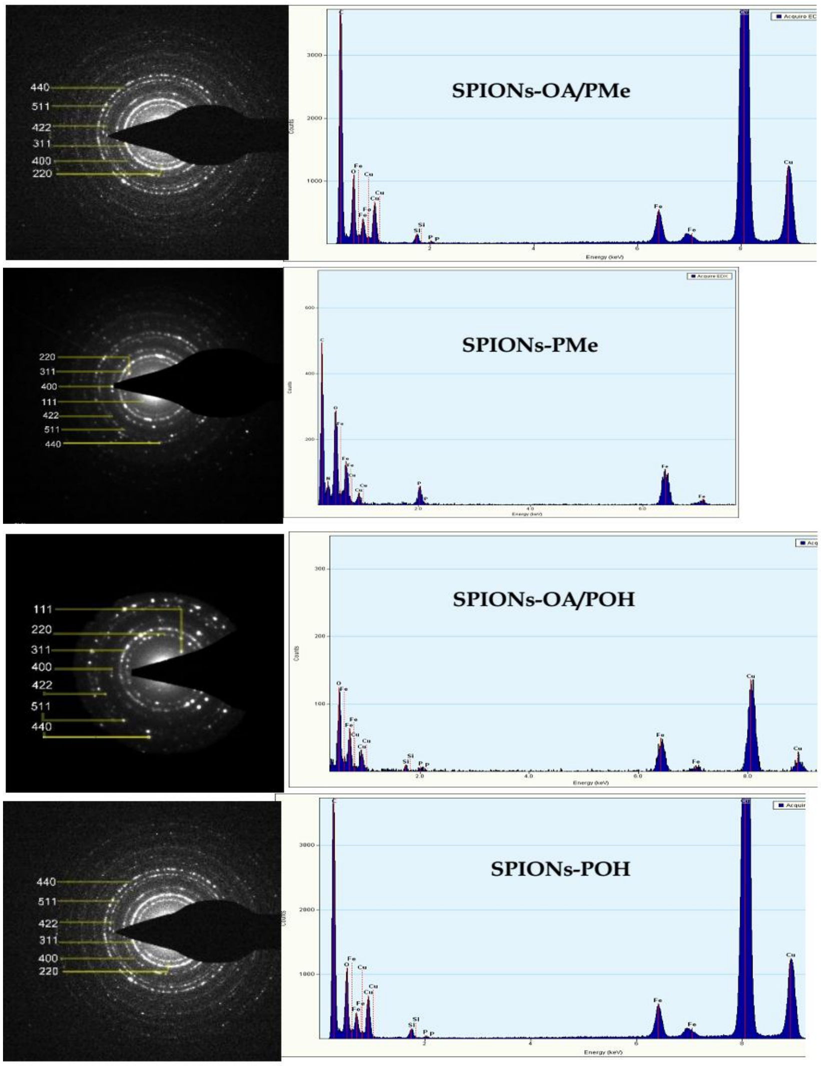


**Figure S2.** SEAD and corresponding EDX analyses for water-dispersible SPIONs. SAED measurements on the TEM samples are on the left-hand side, and the corresponding EDX graphs on the right. For better comparison, the first row pertains to the ligand exchange protocol with phosphonate-TEG-Me and the second for direct conjugation with this ligand, and the third and last row albeit using phosphonate-TEG-OH.

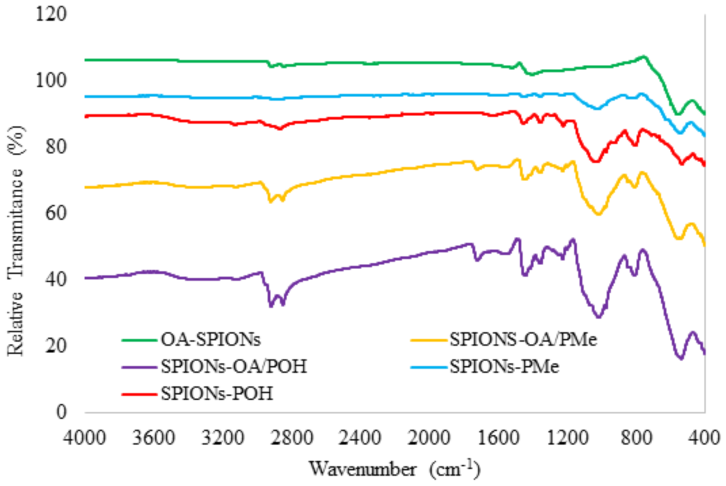


**Figure S3.** Fourier transform infrared spectroscopy (FT-IR) spectra overlay of SPIONs.

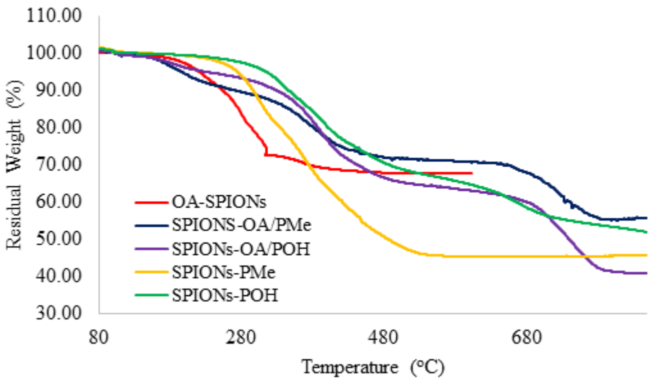


**Figure S4.** Thermogravimetric analysis (TGA) spectra overlay for SPIONs.

**Table S1.** Weight loss for SPIONs calculated from TGA.

| Species | Weight Loss Attributed to OA (%) | Weight Loss Attributed to Phosphonate Ligand (%) | Total Weight Loss (%) |
|---|---|---|---|
| OA-SPIONs | 22 | - | 22 |
| SPIONs-OA/PMe | 16 | 28 | 44 |
| SPIONs-PMe | - | 56 | 56 |
| SPIONs-OA/POH | 23 | 34 | 57 |
| SPIONs-POH | - | 48 | 48 |

**Table S2.** Percent ligand conjugation for SPIONs containing Oleic Acid calculated from UV-Vis analyses.

| Species | Peak Absorbance Maximum (au) | Ligand Remaining Unconjugated (%) | Maximal Conjugation (%) |
|---|---|---|---|
| Phosphonate-TEG-Me | 1.80 | - | - |
| SPIONs-OA/PMe Supernatant | 1.38 | 76.6 | 23.4 |
| Phosphonate-TEG-OH | 1.56 | - | - |
| SPIONs-OA/POH Supernatant | 0.92 | 58.9 | 41.1 |

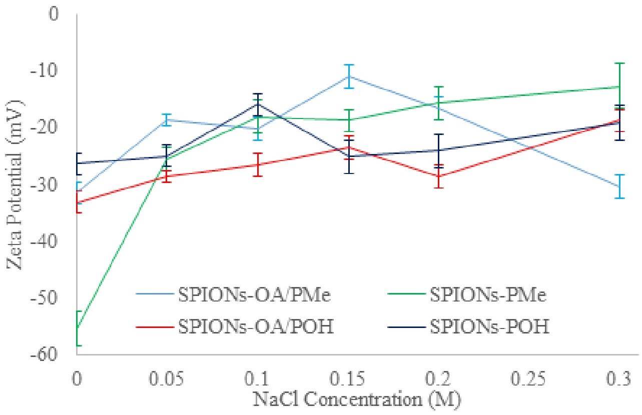


**Figure S5.** Zeta potential values at various NaCl concentrations for water-dispersible SPIONs.

**Table S3.** Uptake of water-dispersible SPIONs in bEnd.3 cells after 24 h.

| SPIONs Species | Treated Cellular Concentration (pg/Cell) | Cellular Uptake (pg/Cell) | % SPIONs Uptaken by Cells |
|---|---|---|---|
| SPIONs-OA/PMe | 243.5 ± 12 | 183.8 ± 18 | 75.5 |
| SPIONs-PMe | 314.7 ± 22 | 247.8 ± 27 | 77.9 |
| SPIONs-OA/POH | 179.4 ± 16 | 144.2 ± 25 | 80.4 |
| SPIONs-POH | 230.4 ± 15 | 209.2 ± 16 | 90.8 |

## References

1. Brunet, E.; Juanes, O.; Jimenez, L.; Rodriguez-Ubis, J.C. Click-chemistry-based bis-triazolylpyridine diphosphonate ligand for the sensitized luminescence of lanthanides in the solid state within the layers of gamma-zirconium phosphate. *Tetrahedron Lett.* **2009**, *50*, 5361–5363.
2. Voicu, R.; Boukherroub, R.; Bartzoka, V.; Ward, T.; Wojtyk, J.T.C.; Wayner, D.D.M. Formation, characterization, and chemistry of undecanoic acid-terminated silicon surfaces: Patterning and immobilization of DNA. *Langmuir* **2004**, *20*, 11713–11720.
3. Kitto, H.J.; Schwartz, E.; Nijemeisland, M.; Koepf, M.; Cornelissen, J.J.L.M.; Rowan, A.E.; Nolte, R.J.M. Post-modification of helical dipeptido polyisocyanides using the 'click' reaction. *J. Mater. Chem.* **2008**, *18*, 5615–5624.
4. Yu, X.; Yang, X.; Horte, S.; Kizhakkedathu, J.N.; Brooks, D.E. A pH and thermosensitive choline phosphate-based delivery platform targeted to the acidic tumor microenvironment. *Biomaterials* **2014**, *35*, 278–286.
5. Turp, D.; Wagner, M.; Enkelmann, V.; Mullen, K. Synthesis of nanometer-sized, rigid, and hydrophobic anions. *Angew. Chem. Int. Ed. Eng.* **2011**, *50*, 4962–4965.
6. Florian, A.; Mayoral, M.J.; Stepanenko, V.; Fernandez, G. Alternated stacks of nonpolar oligo(p-phenyleneethynylene)-BODIPY Systems. *Chem. Eur. J.* **2012**, *18*, 14957–14961.
7. Nierengarten, J.F.; Zhang, S.; Gegout, A.; Urbani, M.; Armaroli, N.; Marconi, G.; Rio Y. Synthesis and optical properties of isomeric branched π-conjugated systems. *J. Org. Chem.* **2005**, *70*, 7550–7557.

© 2016 by the authors; licensee MDPI, Basel, Switzerland. This article is an open access article distributed under the terms and conditions of the Creative Commons by Attribution (CC-BY) license (http://creativecommons.org/licenses/by/4.0/).
